# Brian Hare and Vanessa Woods, Review of *Survival of the Friendliest*

**DOI:** 10.1093/emph/eoab002

**Published:** 2021-01-25

**Authors:** Paul W Turke

**Affiliations:** Turke and Thomashow Pediatrics, 7444 Dexter-Ann Arbor Rd, Suite A, Dexter, MI 48130, USA, Tel: +1 734 408 4182

*Survival of the Friendliest*, by husband-and-wife team Brian Hare and Vanessa Woods, is a well-written, well-argued exploration of the selection pressures and resulting adaptations that help to explain why we treat some groups of strangers with kindness and others with cruelty. The book taught me quite a lot about a topic I thought I knew well, and I can recommend it highly for that alone.

It is based on, and extends, the self-domestication theory that Hare helped to develop along with two mentors, Richard Wrangham and Michael Tomasello [1]. The gist is, self-domestication began in earnest in our species sometime after the emergence of anatomically modern humans around 200 000 years ago, when individuals less prone to ‘reactive aggression’ became preferred social partners. This facilitated the formation of larger, more complex social groups, and because cooperation en masse confers wide-ranging competitive advantages (including technological advances), participants increased their reproductive success, and humans thereby evolved to be friendly, gregarious organisms.

I am a fan of the self-domestication theory, but I think it’s worth noting that it did not appear out of thin air. Darwin was a devoted student of domestication, and had this to say: ‘… those animals which were benefited by living in close association, the individuals which took the greatest pleasure in society would best escape various dangers; whilst those that cared least for their comrades and lived solitary would perish in greater numbers’ (p.80) [2]. And Richard Alexander and Katharine Noonan, writing on how humans evolved their numerous distinctive attributes [3], identify some of the same traits and themes that standout in *Survival of the Friendliest*, including physical characteristics such as a longer juvenile life, greater infantile helplessness (altriciality), relative hairlessness and social characteristic such as extensive extrafamilial nepotism, extensive organized intergroup aggression (war) and polities of thousands or millions of nuclear families.

Among the highlights of the book for me was the description and interpretation of Nikolai Belyaev’s experiment on foxes. It was conducted while he was in exile in Siberia during Stalin’s reign of terror. Stalin, was not fond of genetics or geneticists, but Belyaev, once removed from the watchful eyes of the authorities, was able to design and run an experiment on foxes that rapidly selected for markedly increased friendliness toward humans. The selective pressure also resulted in a suite of traits that included smaller teeth, shorter snouts, curled tails and an increased window for socialization with humans.

Equally interesting is Hare and Woods’ description of the evolution of dogs from wolves. They suggest that members of a wolf pack that happened to be less fearful of humans than was typical were on that account more willing to hang out on periphery of early human encampments. This purportedly gave them access to discarded consumables, including nutrient-rich human excrement, which in turn yielded them a survival and reproductive advantage over their more fearful brethren. Iterated, we got dogs, and perhaps an explanation for why one of my good friends, a particularly lovable Labrador retriever, is so intent on eating the goose poop we encounter while on walks.

I was also intrigued by the many connections the authors were able to make between current events and their account of how friendliness evolved. I was initially wary of this line of argument because I, like many in the United States, am suffering from political fatigue at the close of 2020. Ultimately, however, Hare and Woods managed to be constructive and balanced in their political musings—antifa and the alt-right were both held up as the unfriendly examples of sociality that they are.

For most of the book readers are free to assume that friendliness is entirely a good thing. Hare and Woods eventually reveal, however, that the same emotional and cognitive abilities that allow and guide our large-scale friendliness also set us up to be ruthlessly aggressive toward perceived outside competitors. My favorite descriptive illustration is that of a mother bear that is a great friend to her cubs, but a homicidal maniac when confronted by an apparent threat to their wellbeing. There are two lessons in this: (i) a bear’s affection and commitment to her cubs is what fuels her viciousness toward assumed aggressors; (ii) we are like bears, but on a larger scale and with nuclear weapons—which is a dismal thought that would make for a dismal conclusion to the book.

Fortunately, they don’t conclude on this note; instead, they end *Survival of the Friendliest* with a glimmer of hope, and a plan to achieve it. In their words, ‘We are at our most tolerant when the architecture of our society facilitates tolerance… [And so] we need to design the spaces we live in so we can meet each other without being afraid, disagree without being disagreeable, and make friends with those least like ourselves’ (p.185). They give more details, but you get the idea.

Now for some agreeable disagreement. *Survival of the Friendliest* takes on broad and complex questions, which I applaud, but doing so almost guarantees that some will be left incompletely answered—perhaps by design because bringing up too many caveats and peripheral issues can detract from the main message. However, there is an important question that I wish Hare and Woods would have addressed. Why is mayhem and murder within human groups so common, even among those comprised of close relatives? As a pediatrician, I am all-too-aware of how overworked Child Protective Services is, and my wife, Laura Betzig, a historian, has for decades regaled me with countless accounts of in-group atrocities: ‘et tu Brute?’

If I were to attempt to explain why successions in ancient Rome (and nearly every other empire) have been redolent with the spilled blood of relatives, I would reference kin selection theory, but in doing so I would encounter a problem similar to the one I’ve just raised for the Hare and Woods thesis. Under kin selection we expect to find, and do find, that groups comprised of closely related individuals usually will be more cooperative (internally) than groups comprised of unrelated, or distantly related, individuals. However, there are exceptions that require explanation, which is what we get when we broaden our approach to include, not just relatedness, but also the specific costs and benefits that accrue to individuals from their social choices [4]. In doing this, it becomes clear that murdering a brother or a cousin to assure one’s own ascension to the throne—which generally comes with exclusive access to the imperial harem—is an action that likely increases reproductive success. Thus, kin selection theory is not all that troubled by the fact that kin often kill each other.

*Survival of the Friendliest* can be ‘rescued’ in a similar way from the challenge posed by the fact there is a lot of in-group violence in the world (a fact that at first might seem to be wholly incompatible with their argument). The mechanisms Hare and Woods have identified that prompt and guide large-scale friendliness make good sense, and they have convinced me of their general validity, but, as in kin selection, predicting social outcomes is challenging given all the complicated costs and benefits. Our evolved tendencies to treat in-group members with kindness and generosity need to be considered along with the many different costs and benefits that bear on social interactions. Sometimes they’ll add up to in-group cooperation, other times, not so much.

In conclusion, *Survival of the Friendliest* asks big, important questions, and solves some of them. And, in case you are wondering why it’s being reviewed in an evolutionary medicine journal, well, violence isn’t healthy, and understanding the two sides of friendliness points the way to a cure.
